# Galvanic Vestibular Stimulation (GVS) Augments Deficient Pedunculopontine Nucleus (PPN) Connectivity in Mild Parkinson's Disease: fMRI Effects of Different Stimuli

**DOI:** 10.3389/fnins.2018.00101

**Published:** 2018-02-28

**Authors:** Jiayue Cai, Soojin Lee, Fang Ba, Saurabh Garg, Laura J. Kim, Aiping Liu, Diana Kim, Z. Jane Wang, Martin J. McKeown

**Affiliations:** ^1^Department of Electrical and Computer Engineering, University of British Columbia, Vancouver, BC, Canada; ^2^School of Biomedical Engineering, University of British Columbia, Vancouver, BC, Canada; ^3^Pacific Parkinson's Research Centre, Vancouver, BC, Canada; ^4^Division of Neurology, Department of Medicine, University of Alberta, Edmonton, AB, Canada; ^5^School of Electronics and Applied Physics, Hefei University of Technology, Hefei, China; ^6^Department of Medicine (Neurology), University of British Columbia, Vancouver, BC, Canada

**Keywords:** vestibular stimulation, pedunculopontine nucleus, functional connectivity, Parkinson's Disease, non-invasive neuromodulation, fMRI

## Abstract

Falls and balance difficulties remain a major source of morbidity in Parkinson's Disease (PD) and are stubbornly resistant to therapeutic interventions. The mechanisms of gait impairment in PD are incompletely understood but may involve changes in the Pedunculopontine Nucleus (PPN) and its associated connections. We utilized fMRI to explore the modulation of PPN connectivity by Galvanic Vestibular Stimulation (GVS) in healthy controls (*n* = 12) and PD subjects even without overt evidence of Freezing of Gait (FOG) while on medication (*n* = 23). We also investigated if the type of GVS stimuli (i.e., sinusoidal or stochastic) differentially affected connectivity. Approximate PPN regions were manually drawn on T1 weighted images and 58 other cortical and subcortical Regions of Interest (ROI) were obtained by automatic segmentation. All analyses were done in the native subject's space without spatial transformation to a common template. We first used Partial Least Squares (PLS) on a subject-by-subject basis to determine ROIs across subjects that covaried significantly with the voxels within the PPN ROI. We then performed functional connectivity analysis on the PPN-ROI connections. In control subjects, GVS did not have a significant effect on PPN connectivity. In PD subjects, baseline overall magnitude of PPN connectivity was negatively correlated with UPDRS scores (*p* < 0.05). Both noisy and sinusoidal GVS increased the overall magnitude of PPN connectivity (*p* = 6 × 10^−5^, 3 × 10^−4^, respectively) in PD, and increased connectivity with the left inferior parietal region, but had opposite effects on amygdala connectivity. Noisy stimuli selectively decreased connectivity with basal ganglia and cerebellar regions. Our results suggest that GVS can enhance deficient PPN connectivity seen in PD in a stimulus-dependent manner. This may provide a mechanism through which GVS assists balance in PD, and may provide a biomarker to develop individualized stimulus parameters.

## Introduction

Falls in older adult populations are a significant cause of morbidity and mortality (Cameron et al., 2012) with non-fatal injuries initiating a vicious cycle leading to a fear of falling, social isolation, loss of independence, deconditioning, and a significantly greater use of health care services (Stevens et al., 2006; Williams et al., 2006).

In Parkinson's Disease (PD), gait disturbances such as decreased stride length and gait variability are associated with increased risk of falls. Balance and gait deficits in PD are frequently refractory to therapy (Azevedo Coste et al., 2014; Perez-Lloret et al., 2014) and may be actually worsened by pharmacological and surgical interventions (Bloem et al., 1996), making falls a significant source of morbidity in PD (Schaafsma et al., 2003). Freezing of gait (FOG) is a syndrome normally seen in advanced PD and can occur when subjects are either on or off medication. FOG may be partly due to a failure to adequately scale amplitudes for the intended movement (Chee et al., 2009) and/or defective motor programming setting by the Supplemental Motor Area (SMA) and its maintenance by the basal ganglia, leading to a mismatch between intention and automation (Chee et al., 2009).

Cognitive and motor function must be carefully integrated to execute gait. Dysfunction of the basal ganglia in PD results in impaired motor control of skilled voluntary movements (Magrinelli et al., 2016) and movements become excessively slow and underscaled in size (Benecke et al., 1987). Biochemically, imbalance in multiple neurotransmitters (including but not limited to dopamine, acetylcholine, and GABA) is seen not only in basal ganglia and motor structures, but also limbic circuitries (Ondo and Hunter, 2003; Perez-Lloret and Barrantes, 2016). Balance disturbance and falls in PD may be more related to disruption in cholinergic rather than dopaminergic neurotransmission (Bohnen et al., 2009).

A key part of the subcortical cholinergic system is the pedunculopontine nucleus (PPN), which appears critically involved in gait disturbances in PD (Mazzone et al., 2005; Androulidakis et al., 2008; Acar et al., 2011), as PPN neuronal loss is evident in PD (Rinne et al., 2008) (Note that although we refer to the “PPN” throughout this manuscript, at the resolution of the imaging used here, it would perhaps be more accurate to refer to this region as the “mesencephalic locomotor region” as it likely includes the cuneiform nucleus—however, we use PPN as this terminology is consistent with much prior literature (e.g., Mazzone et al., 2005; Androulidakis et al., 2008; Acar et al., 2011).

Connectivity to/from the PPN appears critical for FOG in PD (Fling et al., 2014). Structural deficits in connectivity are evident between basal ganglia-PPN and other tracts in FOG (Fling et al., 2014; Vercruysse et al., 2015). DTI tractography obtained with 3T MR imaging in PD patients with FOG has demonstrated asymmetrically decreased connectivity between the PPN and the SMA, compared to PD subjects without FOG (Fling et al., 2014). FOG is also associated with diffuse white matter damage involving major cortico-cortical, corticofugal motor, and several striatofrontal tracts with DTI (Vercruysse et al., 2015). In addition to structural/anatomical connectivity, advanced neuroimaging techniques have enabled the studies of functional connectivity, which refers to the statistical temporal dependences between anatomically separated brain regions, to reveal the functional communication in the brain. Functional imaging studies (e.g., fMRI) have reported increased activity or altered connectivity during gait visualization in the midbrain locomotion centers between FOG episodes (Hanakawa et al., 1999), possibly reflecting compensatory mechanisms which might be overwhelmed with stress by turning or multitasking (Shine et al., 2013). Moreover, resting state functional magnetic resonance imaging (rs-fMRI) has allowed the inference of functional connectivity by measuring the level of spontaneous co-activation between fMRI time courses of brain regions recorded during rest. *In vivo* functional connectivity studies with rs-fMRI suggest that FOG patients may have significantly altered connectivity between PPN-SMA (Fling et al., 2014), which might reflect a maladaptive compensatory mechanism.

While the PPN has most often been investigated in PD in the context of FOG, it is unclear if altered PPN activity is present in non-FOG PD patients. Surgical targeting of the PPN is usually reserved for people with FOG resulting in significant impairment. Yet, even in early stages of the disease, there are a number of ways in which Parkinsonian gait is different from controls. While mildly affected PD patients can usually perform simple straight-line walk tasks without difficulty, they experience difficulties with turning, and when performing simultaneous motor or cognitive tasks (dual tasks), and/or crossing obstacles (Camicioli et al., 1998; Bond and Morris, 2000). They may have an abnormal gait pattern characterized by a shortened stride length, increased stride variability, and reduced walking speed (Morris et al., 2001; Buckley et al., 2008).

Ways to modulate PPN activity and connectivity have proven elusive. Acetylcholinesterase inhibitors may affect the PPN but such effects are likely to be modest. PPN Deep Brain Stimulation (DBS) has been shown to (inconsistently) improve gait difficulties in PD (Mazzone et al., 2005; Peppe et al., 2010; Acar et al., 2011; Hamani et al., 2011; Tykocki et al., 2011; Wilcox et al., 2011). However, the PPN tends to be spatially diffuse and is difficult to visualize on standard T1-weighted images, making electrode placement for DBS therapy difficult. Another potential way to modulate PPN is through the vestibular system, as PPN neurons tend to be highly vestibular-responsive (Aravamuthan and Angelaki, 2012). Galvanic vestibular stimulation (GVS) is a non-invasive technique that activates vestibular afferents to the thalamus and also the basal ganglia (Stiles and Smith, 2015) which in turn are directed to the PPN (Visser and Bloem, 2005), possibly explaining why GVS may positively impact posture/standing balance in PD (Kataoka et al., 2016). A few studies have demonstrated that noisy GVS improved postural and balance responses (Pal et al., 2009; Samoudi et al., 2015) as well as motor deficits in PD (Yamamoto et al., 2005; Pan et al., 2008; Lee et al., 2015a,b). These studies have speculated that noisy vestibular input may have improved information flow through the basal ganglia via *stochastic facilitation* (SF). SF is a phenomenon observed in a non-linear system where stochastic biological noise paradoxically increases sensitivity of a system to detect a weak stimulus possibly resulting in functional benefits (Mcdonnell and Ward, 2011). In addition to noisy stimuli, sinusoidal stimuli have been suggested as a means to activate steady-state, as opposed to transient balance responses that would be induced with pulsed stimuli (Latt et al., 2003). Sinusoidally oscillating stimuli may also activate irregular vestibular afferents (Gensberger et al., 2016) relying on voltage dependent K-channels (Eatock and Songer, 2011). While a couple of fMRI studies have shown sinusoidal GVS modulated activations in various brain regions (Della-Justina et al., 2014; Lee et al., 2016), GVS's influence on the PPN has not yet been investigated. A recent study in healthy older adults (*n* = 20) found that noisy GVS resulted in sustained reduction in Centre of Pressure (COP) parameters, such as velocity, and Root Mean Square (RMS) (Fujimoto et al., 2016). The mechanisms of this reduction was speculated to be on the basis of induced synaptic plasticity in the vestibular nuclei and the flocculus of the cerebellum, but effects on the PPN were not considered (Fujimoto et al., 2016).

Given the non-invasive, and potentially portable nature of GVS, we wished to determine if PPN connectivity could be modulated in mildly-affected PD subjects who may demonstrate reduced stride length for example, and thus may be at increased risk for falls, but did not exhibit FOG. Thus, in this study, we investigated whether or not functional connectivity between the PPN and other brain regions could be reliably assessed, whether or not these connections were significantly modulated by GVS, and if the connectivity was modulated in a stimulus-specific manner. Since *a priori* knowledge about functional connectivity to/from the PPN at the spatial and temporal resolution afforded by fMRI is unknown, this was essentially an exploratory approach. Careful care was taken to detect robust activation from PPN structures by analyzing the data in native space (without registration to a template) and utilizing subject-specific weightings of voxels within the PPN region. We demonstrated that PPN connectivity is sensitive to vestibular stimulation in PD in a stimulus-dependent manner.

## Methods

### Subjects

Twenty three PD patients (see Table 1 for the clinical information) and 12 age-matched healthy controls [5 females; age: 63.3 ± 10.4 (mean ± standard deviation)] participated in the study. The PD patients had mild to moderate PD (Hoehn and Yahr stage I–III) (see Table 1) and were scanned at the on-medication state. All participants were recruited from the Pacific Parkinson's Research Centre (PPRC) at the University of British Columbia (UBC) and provided written, informed consent prior to participation. All studies were approved by the UBC Ethics Review Board. The data were collected in two experiments: in the first group GVS was assessed both ON and OFF L-dopa medication and has been partially reported in a separate short report (Lee et al., 2016), and the second group were only assessed in the ON medication state. Only the ON medication studies from the first group are reported here. In the first group, UPDRS scores Part III were assessed in the OFF medication state, while in the second group UPDRS scores were assessed in the ON medication state. A regression model was used to control for these differences (described below).

**Table 1:** Clinical information on PD patients.

**Characteristics**	**Statistics (mean ± standard deviation)**
Age	66.4 ± 7.0
Sex	17 males, 6 females
UPDRS motor score	22.3 ± 12.4
UPDRS assessed during on/off medication	13 on-medication, 10 off-medication
Hoehn and Yahr stage	1.9 ± 1.0
L-dopa equivalent daily dose (LEDD)	988.8 ± 798.9

### GVS

Digital signals of the GVS stimuli were first generated on a PC with MATLAB (MathWorks, MA, USA) and were converted to analog signals via a NI USB-6221 BNC digital acquisition module (National Instruments, TX, USA). The analog command voltage signals were then subsequently passed to a bipolar, constant current stimulator (DS5 model, Digitimer Ltd., U.K.). The DS5 constant current stimulator was isolated in the console room with the output cable leading into the scanning room through a waveguide. Along the twisted coaxial output cable, four inductance capacity filters spaced 20 cm apart and tuned for the Larmor frequency (128 MHz) were custom-built. Near the subject, high-resistance radiotranslucent carbon-fiber leads (Biopac Inc., Montreal, Canada) were connected to pre-gelled Ag/AgCl electrodes that were MR-compatible (Biopac Inc., Montreal, Canada). For bilateral stimulation, an electrode was placed over the mastoid process behind each ear. Since the GVS stimuli are alternating current (AC), the anode and cathode are not fixed on one side (as for DC) but they are alternating. The order of GVS condition was kept consistent to be rest, noisy GVS, and sinusoidal GVS across all the participants. The potential caveat of keeping the sequence the same is the case where there are any post-stimulation effects. To avoid such confounding effects, we allowed a 2-min break between the two GVS conditions. To the best of our knowledge, after-effects of GVS on cortical activation have not yet been investigated. However, we think that the break time was sufficient to avoid after effects based on literature on after-effects of transcranial alternating current stimulation (Strüber et al., 2015).

Since individuals have an inherently subjective perception of GVS, prior to scanning, we determined the individual sensory threshold level (cutaneous sensation at the electrode site) utilizing systematic procedures used in prior GVS studies (Hummel et al., 2005; Wilkinson et al., 2008; Utz et al., 2011). We delivered two different types of stimuli at 90% of the individual threshold level: noisy and sinusoidal. The noisy stimulus was zero-mean with 1/f-type power spectrum between 0.1 and 10 Hz and the sinusoidal stimulus was a 1 Hz sine wave.

### MRI

Resting state data were collected on a 3 Tesla scanner (Philips Achieva 3.0T R3.2; Philips Medical Systems, Netherlands) equipped with a head-coil. During the scanning, all the subjects were instructed to be awake with eyes closed. Blood oxygenation level-dependent (BOLD) contrast echo-planar (EPI) T2^*^-weighted images were taken with the following specifications with a repetition time of 1,985 ms, echo time of 37 ms, flip angle 90°, field of view 240.00 mm, matrix size 128 × 128, and with pixel size 1.9 × 1.9 mm. The duration of each functional run was 8 min for rest condition and 5 min for GVS condition with noisy and sinusoidal stimulus, respectively. As stated above, the order of functional runs was rest, noisy GVS and sinusoidal GVS, and it was kept consistent across all subjects. We allowed 2 min gaps after the noisy stimulus to account for possible post-stimulation effects.

**Figure 1:**
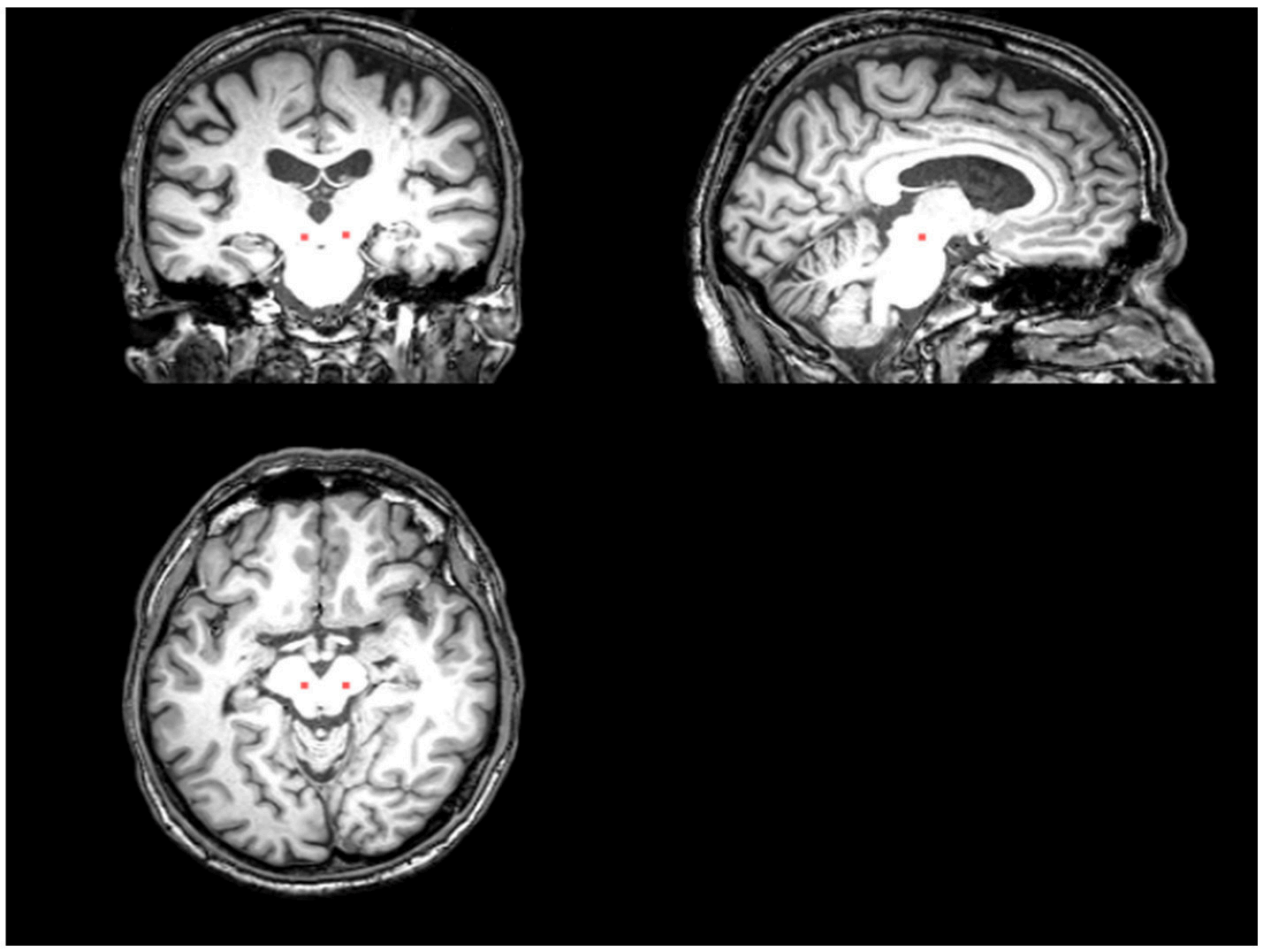
The placement of the PPN VOI on the T1 sequence. The red area represents where the PPN VOI is placed.

An ROI presumed to include the PPN and cuneiform nucleus was drawn manually on the T1 sequence at the level of the superior cerebellar decussation between medial lemniscus and superior cerebellar peduncle (Aravamuthan et al., 2007; Zrinzo et al., 2008) (Figure 1). T1 and the fMRI data were registered via FLIRT (with “Boundary-Based Registration” option) in FSL (Smith et al., 2004). The PPN voxels drawn on the T1 were then registered to the fMRI data by applying the inverse transformation calculated by registering the fMRI to the T1 weighted image. After registration, we included neighboring voxels around PPN voxels in our analysis to account for possible partial volume effects.

### Resting state functional MRI (rs-fMRI) preprocessing

The acquired fMRI data were preprocessed using both AFNI and SPM8 software packages. On the whole brain, several preprocessing steps from the AFNI software package were performed. These included despiking, slice timing correction, and 3D isotropic correction (3 mm in each dimension). While the subjects were asked to keep the head still during the scanning session, some head movements occurred during the acquisition process. Motion correction using rigid body alignment was performed to correct for any major head motion during the scan. Besides the fMRI scans, we also collected a T1-weighted structural scan of each of the participants. FreeSurfer was performed on the T1-weighted scans to get the different ROI masks in the T1 space. Each of the subjects' structural scans was then registered to the fMRI scan using rigid registration. This registration step provided us with the FreeSurfer segmented ROI mask in the fMRI space. All analysis was done in the individual fMRI space rather than transforming all fMRI data to a common template. This was done to prevent introducing any unwanted distortions in the fMRI data by registering it to a common template. In the next step, several sources of variance such as head-motion parameters, their temporal derivatives and their squares, white-matter signal, CSF signal were removed using nuisance regression. The fMRI signal was then detrended, and any linear or quadratic trends in the fMRI signal were removed. The signal was then iteratively smoothed until it reached 6 FWHM of smoothness. Finally, bandpass filtering was performed to retain the signal between the recommended frequencies of interest (0.01–0.08 Hz).

Since the brainstem can move independently from the rest of the brain, motion correction on the whole brain motion estimates may not be ideal. Therefore, a separate motion correction of the brainstem was performed. First, the brainstem mask was generated using FreeSurfer on the T1-weighted image of the same subject. The mask was then transferred over to the fMRI using registration as mentioned before. The registered mask was then dilated using a spherical structuring element of radius 3 to incorporate for any errors in the segmentation and registration process. The motion within the brainstem was then corrected independently using the SPM toolbox.

### Brain region selection

In this study, we included 58 ROIs automatically segmented by FreeSurfer as shown in Table 2. Two PPN ROIs were manually drawn on the T1-weighted images to include the PPN on each side, namely left PPN, and right PPN, respectively. When assessing the functional connectivity between PPN and other brain regions, we first utilized Partial Least Squares (PLS) to initially select candidate brain regions that significantly covaried with PPN voxels.

**Table 2:** The 58 ROIs (in addition to the 2 PPN ROIs) used in the analysis.

1	Left-Cerebellum-Cortex
2	Left-Thalamus-Proper
3	Left-Caudate
4	Left-Putamen
5	Left-Pallidum
6	Left-Hippocampus
7	Left-Amygdala
8	Left-Accumbens-area
9	ctx-lh-caudalanteriorcingulate
10	ctx-lh-caudalmiddlefrontal
11	ctx-lh-cuneus
12	ctx-lh-entorhinal
13	ctx-lh-inferiorparietal
14	ctx-lh-inferiortemporal
15	ctx-lh-lateralorbitofrontal
16	ctx-lh-medialorbitofrontal
17	ctx-lh-middletemporal
18	ctx-lh-parahippocampal
19	ctx-lh-paracentral
20	ctx-lh-postcentral
21	ctx-lh-posteriorcingulate
22	ctx-lh-precentral
23	ctx-lh-precuneus
24	ctx-lh-rostralanteriorcingulate
25	ctx-lh-rostralmiddlefrontal
26	ctx-lh-superiorfrontal
27	ctx-lh-superiorparietal
28	ctx-lh-superiortemporal
29	ctx-lh-insula
30	Right-Cerebellum-Cortex
31	Right-Thalamus-Proper
32	Right-Caudate
33	Right-Putamen
34	Right-Pallidum
35	Right-Hippocampus
36	Right-Amygdala
37	Right-Accumbens-area
38	ctx-rh-caudalanteriorcingulate
39	ctx-rh-caudalmiddlefrontal
40	ctx-rh-cuneus
41	ctx-rh-entorhinal
42	ctx-rh-inferiorparietal
43	ctx-rh-inferiortemporal
44	ctx-rh-lateralorbitofrontal
45	ctx-rh-medialorbitofrontal
46	ctx-rh-middletemporal
47	ctx-rh-parahippocampal
48	ctx-rh-paracentral
49	ctx-rh-postcentral
50	ctx-rh-posteriorcingulate
51	ctx-rh-precentral
52	ctx-rh-precuneus
53	ctx-rh-rostralanteriorcingulate
54	ctx-rh-rostralmiddlefrontal
55	ctx-rh-superiorfrontal
56	ctx-rh-superiorparietal
57	ctx-rh-superiortemporal
58	ctx-rh-insula

PLS is a statistical method that explores the predictive models between predictor variables and response variables (Wold, 1985). It constructs a linear regression model by projecting the predictor variables and response variables to a new set of latent variables the covariance of which is maximized. PLS is particularly useful when the predictor variables are highly collinear, or when the number of predictor variables is larger than that of observations, while classical multiple linear regression models will fail in these cases. PLS has been widely used in various fields of chemometrics, social science, bioinformatics, and neuroscience (e.g., Ziegler et al., 2013; Chen et al., 2016).

When applying PLS, we used the 58-ROI dataset as predictor variables, X, and the PPN voxels as response variables, Y, and then tried to predict PPN activity from those 58-ROI time courses.

The general model for PLS is

(1) $$\begin{array}{rcl} X & = & \text{T}P^{T}+\text{ E} \end{array}$$

(2) $$\begin{array}{rcl} Y & = & \text{U}Q^{T}+\text{ F} \end{array}$$

where X is a *t*-by-*m* matrix of ROI data (predictor variables), with *t* corresponding to the number of time points, and *m* (= 58) representing the number of subject-independent (non-PPN) ROIs; Y is a *t*-by-*n* matrix of PPN voxel time courses (response variables), where *n* is the number of PPN voxels (which was subject-dependent); T and U are, respectively, *t*-by-*c* component matrices decomposed from X and Y (T and U are also called X Score and Y Score, respectively), where *c* is the number of components; P is an *m*-by-*c* loading matrix of ROI dataset, and Q is an *n*-by-*c* loading matrix of PPN voxels; and E, F are the *t*-by-*m* and *t*-by-*n* matrices, respectively, representing error terms. Essentially, PLS performs the decompositions of X and Y to maximize the covariance between T and U.

We then interrogated the loadings of the X components (i.e., the columns of P) to determine if they were significantly different from zero across subjects. The same procedure was conducted for both left PPN and right PPN, respectively, and then the union set of the selected regions from left PPN and right PPN was used as the final candidate set of brain regions. In addition, we used the first component of Y (i.e., the first column of Y Score) to represent the PPN signal in the subsequent functional connectivity analyses.

### Functional connectivity analyses

We further performed functional connectivity analyses between the PPN and PLS-derived regions. Functional connectivity measures were obtained by computing the partial correlation coefficients between the represented PPN signal, which was obtained by the PLS analysis, and the averaged time courses of each PLS-derived region. We conducted the functional connectivity analyses on a subject-by-subject and task-by-task basis. Specifically, for each subject, functional connectivity was assessed for each of the three conditions, i.e., rest, noisy and sinusoidal GVS conditions, by taking the time courses for each condition time segment of interest from the PPN and PLS-derived regions and computing the partial correlation coefficients between them. For simplicity, we summed the absolute values of the significant connectivity coefficients from both left and right PPN to get an overall PPN connectivity.

To investigate whether or not functional connectivity between the PPN nuclei and PLS-derived regions was significantly affected by GVS, we calculated overall PPN connectivity differences between GVS on (i.e., noisy/sinusoidal GVS condition) and GVS off (i.e., rest condition). An independent one-sample *t*-test was then performed on the calculated connection coefficient *differences* across subjects, with the null hypothesis that the difference was zero, to determine if significant connectivity changes were induced by GVS.

## Results

### Brain region selection

**Figure 2:**
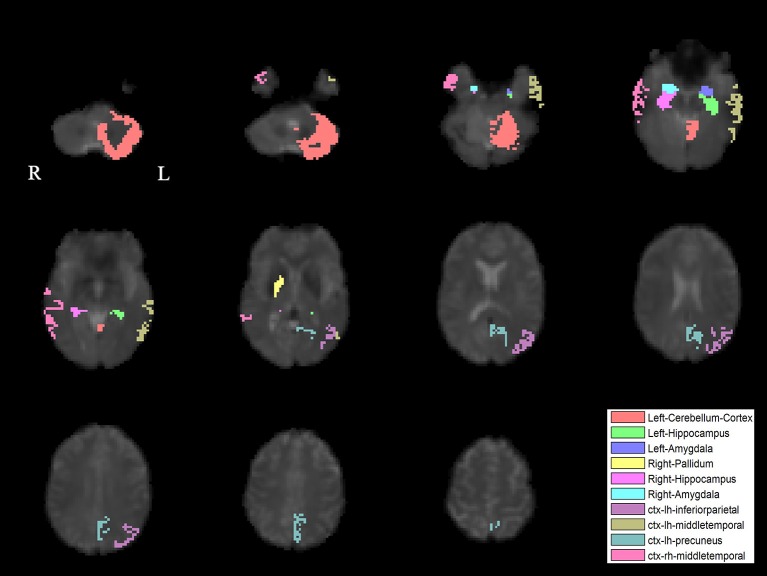
PLS-derived ROIs in the PD group that significantly covaried with PPN voxels. The detected 10 regions, including cerebellum cortex, hippocampus, amygdala, inferior parietal, middle temporal, and precuneus regions on the left, and the pallidum, hippocampus, amygdala, and middle temporal on the right, are marked with different colors.

**Figure 3:**
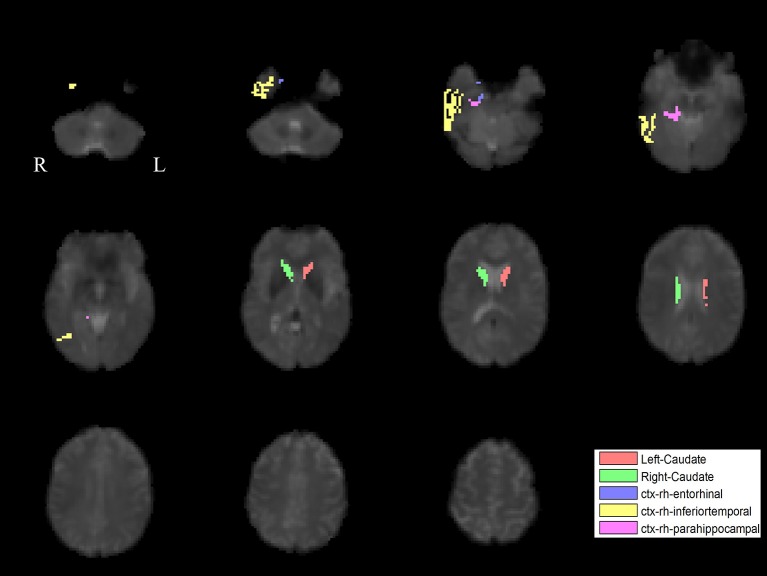
PLS-derived ROIs in the control group that significantly covaried with PPN voxels. The detected five regions, including the caudate on the left, and the caudate, entorhinal cortex, inferior temporal, and parahippocampal regions on the right, are marked with different colors.

The PLS analysis results found 10 ROIs in the PD group and 5 ROIs in the control group that significantly covaried with PPN voxels (*p* < 0.05). In the PD group, the ROIs included the cerebellum cortex, hippocampus, amygdala, inferior parietal, middle temporal, and precuneus regions on the left, and the pallidum, hippocampus, amygdala, and middle temporal on the right (Figure 2). In the control group, the caudate on the left, and the caudate, entorhinal cortex, inferior temporal, and parahippocampal regions on the right were associated with PPN activity (Figure 3).

### Functional connectivity analyses

To determine the effect of the L-dopa equivalent daily dose (LEDD) on connectivity and to correct for the fact that some subjects had their UPDRS assessed off medication, we performed a regression analysis where connection strengths across subjects was the dependent variable, and LEDD, UPDRS score, whether or not the UPDRS was done on or off medication were independent variables. We then evaluated the regression coefficients for the LEDD to determine if it had a significant effect on overall PPN connectivity, which it did not (*p* > 0.05).

**Figure 4:**
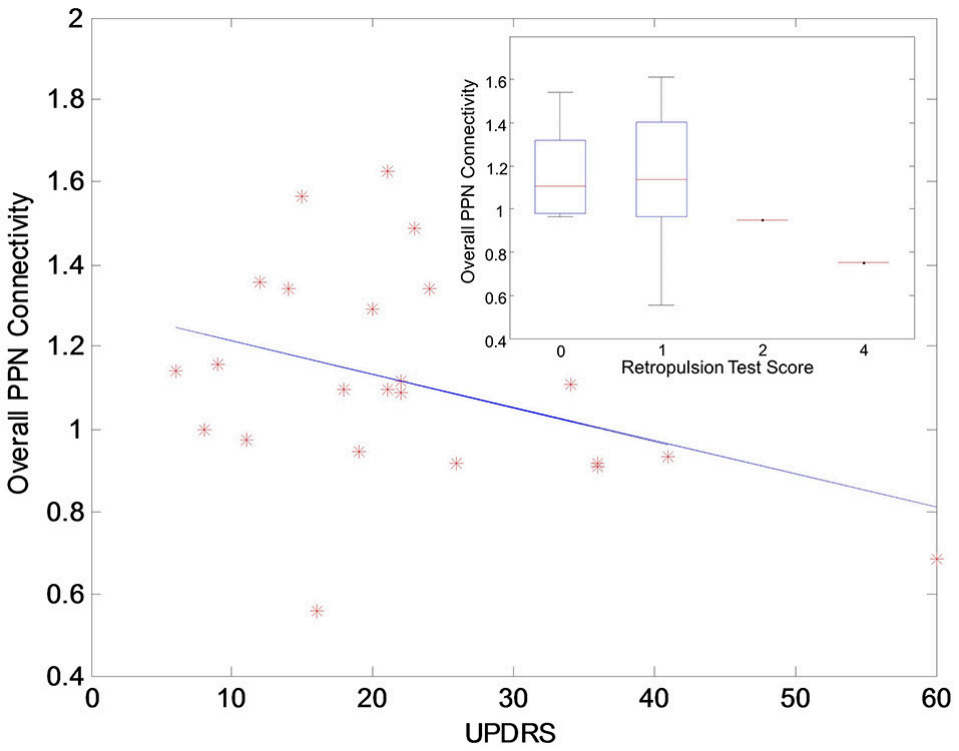
The correlation relationship between the overall PPN connectivity and UPDRS scores in the PD group. The inset shows the relationship between the overall PPN connectivity and the retropulsion test scores from the UPDRS.

**Figure 5:**
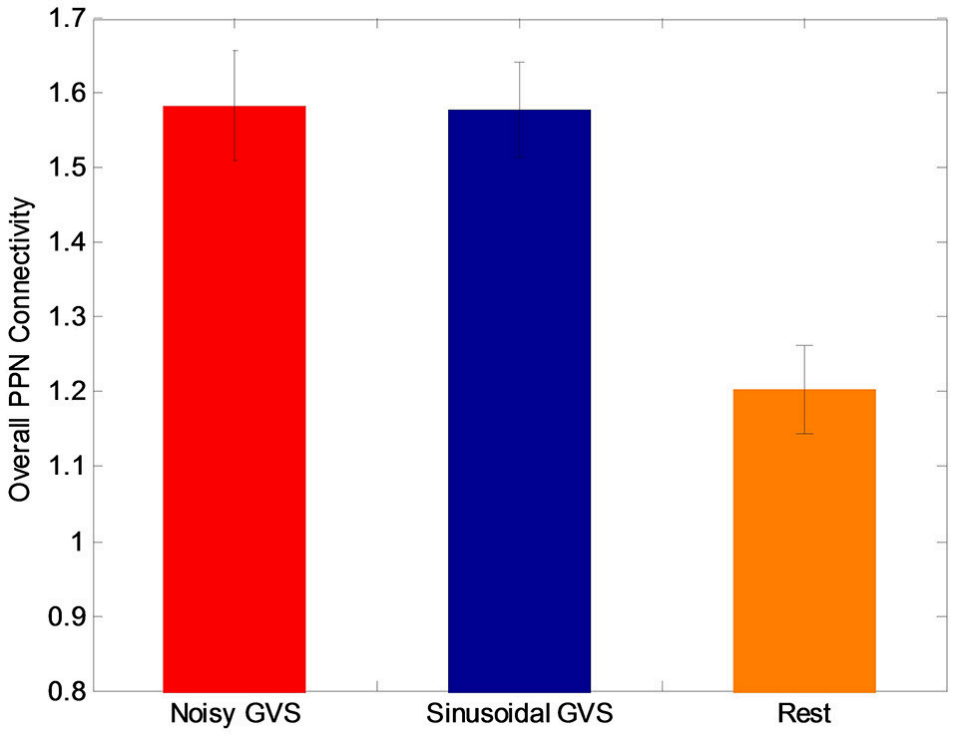
The overall PPN connectivity differences between GVS on (i.e., noisy and sinusoidal GVS condition) and GVS off (i.e., rest condition) in the PD group.

The functional analysis results demonstrated that GVS differently affected overall PPN connectivity in PD and control groups. In the control group, no significant differences in overall PPN connectivity were found between GVS on (i.e., noisy/sinusoidal GVS condition) and GVS off (i.e., rest condition). In the PD group, the overall magnitude of PPN connectivity correlated negatively with UPDRS scores (*r* = −0.39, *p* = 0.035, Figure 4). Both noisy and sinusoidal GVS increased the magnitude of overall PPN connectivity (*p* = 6 × 10^−5^ and 3 × 10^−4^, respectively, Figure 5). Furthermore, in order to determine if overall connectivity of the PPN was particularly related to postural instability, we also compared the connectivity to the retropulsion test score from the UPDRS (Figure 4, inset). Since our emphasis was on early patients, we could not perform a statistical analysis, as there was only 1 subject with a score of 2 and 1 subject with a score of 4. However, as shown in Figure 4, and consistent with overall UPDRS scores, there was a trend toward decreased connectivity with higher retropulsion test scores.

Although both types of stimuli augmented overall PPN connectivity (both positive and negative connectivity as shown in Figure 5), in order to determine if there are differences between the types of stimuli, we performed a *post*-*hoc* analysis to determine which PPN connections were most influential in determining changes in overall connectivity. Specifically, we performed *t*-tests on each connection to determine if the different types of GVS stimuli increased or decreased connectivity between the PPN and other brain regions.

**Figure 6:**
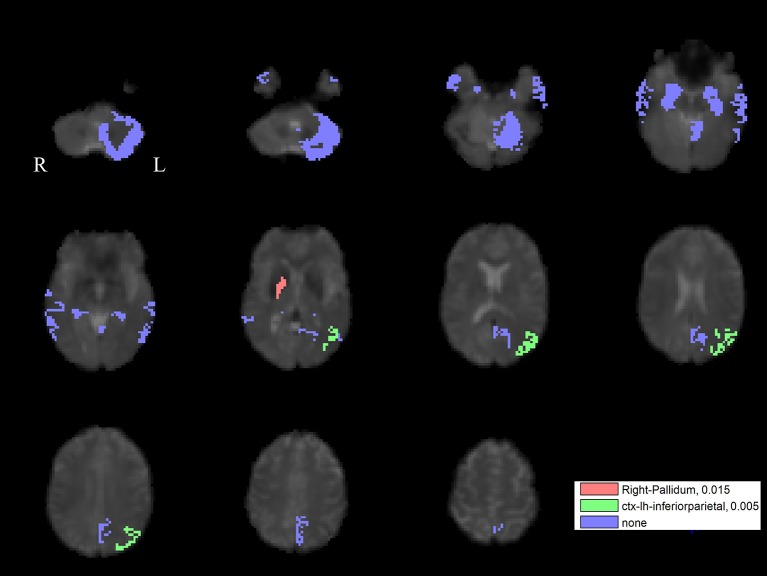
The GVS impact on the connectivity between the left PPN and PLS-derived regions in the PD group. The red areas represent the regions significantly affected by noisy GVS. The green areas represent the regions significantly affected by sinusoidal GVS. The blue areas represent the regions with no significant changes. The corresponding ROI names and significance values are labeled in the figure.

**Figure 7:**
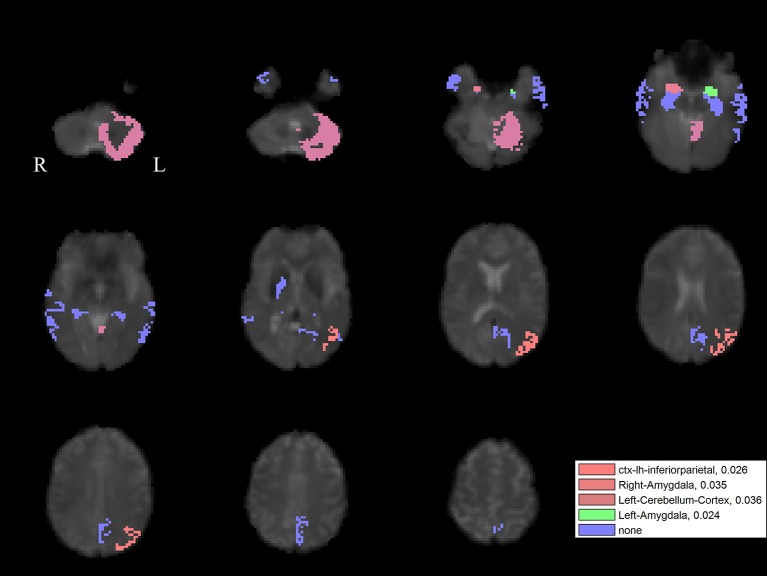
The GVS impact on the connectivity between the right PPN and PLS-derived regions in the PD group. The red areas (with three different color levels indicating the different levels of significance values) represent the regions significantly affected by noisy GVS. The green areas represent the regions significantly affected by sinusoidal GVS. The blue areas represent the regions with no significant changes. The corresponding ROI names and significance values are labeled in the figure.

For the left PPN, noisy GVS decreased connectivity with the right pallidum and sinusoidal GVS increased connectivity with the left inferior parietal region (Table 3 and Figure 6). For the right PPN, noisy GVS decreased connectivity with the left cerebellar cortex, increased connectivity with the right amygdala and increased connectivity with the left inferior parietal region; sinusoidal GVS decreased connectivity with the left amygdala (Table 4 and Figure 7). Note that only the connection between left PPN and left inferior parietal region would survive multiple comparisons.

**Table 3:** Statistics on significant left PPN connectivity changes induced by GVS stimuli in the PD group.

**Connectivity**	**Type of Stimuli**	***t*-value**	***p*-value**
Right pallidum	Noisy GVS	−2.32	0.015
Left inferior parietal	Sinusoidal GVS	2.81	0.005

**Table 4:** Statistics on significant right PPN connectivity changes induced by GVS stimuli in the PD group.

**Connectivity**	**Type of Stimuli**	***t*-value**	***p*-value**
Left cerebellum cortex	Noisy GVS	−1.89	0.036
Right amygdala	Noisy GVS	1.90	0.035
Left inferior parietal	Noisy GVS	2.05	0.026
Left amygdala	Sinusoidal GVS	−2.09	0.024

## Discussion

Balance impairment remains a vexing problem in PD and is associated with considerable morbidity. Pharmacological (particularly dopaminergic) and surgical interventions have had varying degrees of success. Development of novel therapies has also been challenging because the pathophysiology and neuropathological substrates underlying gait disturbances are incompletely known.

To the best of our knowledge, we have shown for the first time that is possible with GVS to non-invasively modulate the functional connectivity in PD subjects between cortical/subcortical ROIs and the PPN—a structure critical for normal supraspinal control of locomotion. This demonstrated alteration in functional connectivity complements previous work examining anatomic connectivity patterns. Anatomically, the PPN has been shown to have connections with various areas such as the vestibular nuclei (Aravamuthan and Angelaki, 2012), deep cerebellar nuclei (Hazrati and Parent, 1992), premotor, supplemental motor (SMA) and primary motor cortices (Aravamuthan et al., 2007), frontal eye fields (Matsumura et al., 2000), thalamic nuclei and basal ganglia nuclei (Charara et al., 1996). Widespread projections involving the PPN include direct glutamatergic inputs from the motor cortex, and GABAergic inputs from substantia nigra pars reticulata (SNr), globus pallidus internus (GPi), subthalamic nucleus (STN), and deep nuclei of cerebellum. Ascending efferent projections target GPi, substantia nigra pars compacta (SNc), and thalamus. Descending efferent projections connect to pontine, medullary reticular formation, and the spinal cord vital for control of muscle tone and locomotion. Additionally, the PPN appears to be important in the initiation, acceleration, deceleration, and termination of locomotion through connections to the basal ganglia and higher cortical regions (Lee et al., 2000).

Our results indicate significant differences in the PPN functional connectivity between PD subjects and controls. This is particularly relevant when noting that none of our PD subjects had FOG, given their relatively mild disease. However, even mild disease is associated with altered gait and our results suggest that PPN functional connectivity patterns change even in the early stages of disease course.

We have shown that both GVS stimuli patterns (noisy and sinusoidal) augment overall deficient PPN connectivity in PD. Our results are consistent with previous studies demonstrating GVS activation of vestibular afferents to basal ganglia (Visser and Bloem, 2005; Stiles and Smith, 2015), which are also directed to the midbrain locomotion network (Peppe et al., 2010). PET studies in humans have also shown activation in the putamen in response to vestibular stimulation (Bottini et al., 1994).

We found differences in PPN network connectivity depending upon the type of stimulus used. Noisy GVS significantly decreased the functional connectivity between the left PPN and right-pallidum in PD. The PPN receives strong inhibitory, GABAergic inputs from the BG nuclei (GPi, STN, and SNr), which have disrupted connectivity in PD (Pahapill and Lozano, 2000). In particular, previous studies suggest that the PPN may be the principal target of pallidal outflow, since more than 80% GPi neurons were found to send axonal branches to both the PPN and thalamus in monkeys (Harnois and Filion, 1982). In PD, the inhibitory GABAergic synaptic activities from the GPi to the PPN is abnormally overactive, which may underlie the akinesia and the gait problems seen in the PD (Pahapill and Lozano, 2000). Taken together, these studies demonstrate the important role of the PPN and pallidal connectivity in motor and gait dysfunctions of PD. We demonstrated that noisy GVS significantly decreased connectivity between the left PPN and right-pallidum in PD (but not normal controls), which suggests that potential benefits of GVS on balance in PD may be partly mediated through attenuation of overactive pallidal inputs to the PPN.

Noisy GVS also decreased connectivity between the right PPN and the left cerebellar cortex. A DWI study found the connectivity of the PPN region with the cerebellum, thalamus, pallidum, and STN (Muthusamy et al., 2007). The cerebellum functions that help control of movement, coordination, and posture (Saab and Willis, 2003) are speculated to be associated with the existence of the pathway with the PPN (Muthusamy et al., 2007). The fact that prior studies have suggested a beneficial effect of noisy GVS (Fujimoto et al., 2016) may indicate that hyperactive PPN-cerebellar connections are partly “normalized” with GVS.

We also found that GVS increased the functional connectivity between the left inferior parietal cortex and the right PPN (with noisy stimuli) and left PPN (with sinusoidal stimuli). Previous studies in non-human primates examining cortical inputs to the pontine nuclei have supported the anatomical and functional relationships between the PPN and the inferior parietal cortex (May and Andersen, 1986; Martinez-Gonzalez et al., 2011). Like the PPN, the left inferior parietal cortex is involved in gait as well as visuospatial information processing, motor planning, and preparation (Caspers et al., 2012). Imagining normal gait activates the left inferior parietal lobule, in addition to the precuneus and bilateral dorsal premotor cortex, the left dorsolateral prefrontal cortex, and the right posterior cingulate cortex (Malouin et al., 2003). The left inferior parietal cortex appears to be especially related to FOG. Gray matter volume in the left inferior parietal region is significantly reduced in PD subjects with FOG patients compared to both PD subjects without FOG and healthy controls (Kostić et al., 2012). Thus, our result that noisy GVS increased the connectivity between the PPN and the left inferior parietal cortex might suggest GVS could improve gait difficulties in PD by augmenting the connectivity.

In the current study, one of the advantages is that we performed analyses keeping each subject's data in their original space without warping the data to a common template. We are frankly skeptical of fMRI studies suggesting robust activation from brainstem structures (e.g., PPN) when data are spatially transformed to a template, given the significant registration errors that can occur to small brainstem nuclei during whole-brain registration (Ng et al., 2009). In addition, we utilized PLS to find the combination of PPN voxels on a subject-by-subject basis that maximally corresponded with other ROIs. In effect, the first column of Q in Equation (2) represents a subject-specific spatial filter to “focus” the activity that maximally covaried with other ROIs.

It is interesting that many of the abnormal connectivities that we detected are lateralized, when balance might be considered a “midline” function. Balance control and gait are asymmetrical in patients with PD, and gait asymmetries have been linked to the pathophysiology of FOG (Boonstra et al., 2014). The symptoms of PD generally show an asymmetric onset and progression and it has been proposed that this may lead to a degree of “unbalanced” motor function, such that FOG is triggered by a breakdown in the bilateral co-ordination underlying the normal timing of gait (Plotnik et al., 2005).

There are a few limitations in our study. We examined a relatively small number of PD patients. However, by carefully selecting the PPN voxels via PLS on a subject-by-subject basis, we expect that we have significantly enhanced our effect size, and thus increasing our statistical power and thus increasing our statistical power. PLS is one of the most widely used blind source separation (BSS) approaches which have largely benefited the neuroscience studies (Ziegler et al., 2013; Chen et al., 2016, 2018; Smith and Nichols, 2018). In the future studies, we are interested to further explore the effective voxel selections using such data-driven approaches. We have shown GVS induced changes in PPN connectivity in people with mild to moderate PD but not in healthy controls. We speculate an “inverted-U” shape of effectiveness of GVS as a function of disease severity: in controls, GVS had minimal effect, in early/moderate PD, it had some effect (shown here), and in severe disease, degeneration in the PPN itself may prevent modulation of its connectivity. Future work is required to further investigate the relationship between disease severity and PPN connectivity and determine the behavioral significance of this altered PPN connectivity. We do note that we found a negative correlation between UPDRS scores and overall PPN connectivity, yet still found robust modulation of connectivity across all of our subjects.

The relation between behavioral gait measures (e.g., gait variability) and ultimate falls risk—the most important issue for people with PD—is an active area of research. Conceivably, previously-described GVS improvements in balance may not ultimately translate into reduced fall risk but such determination would require a prospective trial in the future. We have focused on the PPN because of its possible therapeutic implications, but gait disturbances in PD likely involve several cortical and subcortical structures. For example, PD patients have decreased activity of the SMA during gait (Hanakawa et al., 1999), and PD individuals have diminished pre-movement electroencephalographic potentials originating from the SMA prior to step initiation (Smith et al., 2012). Future studies to assess connectivity changes modified by GVS in other supra-spinal locomotion centers including SMA/pre-SMA may help to guide the development of optimal stimuli on a subject-specific basis.

## Ethics statement

This study was carried out in accordance with the recommendations of TCPS2, UBC Clinical Research Ethics Board with written informed consent from all subjects. All subjects gave written informed consent in accordance with the Declaration of Helsinki. The protocol was approved by the UBC Clinical Research Ethics Board.

## Author contributions

This study is ideated by MM. SL, LK, and DK conducted the experiment and data acquisition. SG and FB contributed to the data preprocessing. JC performed data analysis and manuscript writing. Interpretation of the results was performed by SL, JC, AL, FB, and MM. MM, ZW, and AL contributed to the project supervision and manuscript finalization. All authors have approved the final manuscript.

### Conflict of interest statement

The authors declare that the research was conducted in the absence of any commercial or financial relationships that could be construed as a potential conflict of interest.
